# „Rebound pain“ – von der Definition bis zur Therapie

**DOI:** 10.1007/s00101-022-01120-z

**Published:** 2022-05-05

**Authors:** Timo Streb, Alexander Schneider, Thomas Wiesmann, Jenny Riecke, Ann-Kristin Schubert, Hanns-Christian Dinges, Christian Volberg

**Affiliations:** 1 Klinik für Anästhesie und Intensivtherapie, Universitätsklinikum Gießen und Marburg, Philipps-Universität Marburg, Baldingerstraße, 35043 Marburg, Deutschland; 2 Fachbereich Psychologie, AG Klinische Psychologie und Psychotherapie I, Philipps-Universität Marburg, Gutenbergstraße 18, 35032 Marburg, Deutschland; 3 Klinik für Anästhesie und Intensivtherapie, Diakonie-Klinikum Schwäbisch Hall, Diakoniestraße 10, 74523 Schwäbisch Hall, Deutschland; 4 AG Ethik in der Medizin, Fachbereich 20, Dekanat Humanmedizin, Philipps-Universität Marburg, Baldingerstraße, 35043 Marburg, Deutschland

**Keywords:** Regionalanästhesie, Postoperative Schmerzen, Periphere Nervenblockade, Hyperalgesie, Schmerztherapie, Regional anaesthesia, Postoperative pain, Peripheral nerve block, Hyperalgesia, Analgesia

## Abstract

**Hintergrund:**

Rebound pain als Nebenwirkung einer Regionalanästhesie ist ein übermäßiges Schmerzempfinden nach Abklingen der Wirkung der lokalen Betäubung, welches deutlich über den normalen Wundschmerz nach einer Operation hinausgeht. Dieses Phänomen rückt seit ca. 10 Jahren stärker in den Fokus der Forschung. Die konkreten Ursachen sind bisher unklar, und es existieren auch noch keine zielgerichteten Therapieempfehlungen.

**Ziel der Arbeit:**

Dieser Übersichtsartikel soll der Leserschaft einen Überblick über den aktuellen Stand der Forschung geben. Es werden die Theorien zur Pathophysiologie vorgestellt und Prophylaxe- sowie Behandlungsstrategien erläutert.

**Material und Methoden:**

Für diese Übersichtsarbeit wurden die von 2005 bis Mai 2021 auf PubMed erschienenen Publikationen zum Thema Rebound pain durchgesehen und die Definitionen der Autoren zu Rebound pain sowie die Annahmen zur Pathophysiologie und Therapieempfehlungen zusammengefasst.

**Ergebnisse und Diskussion:**

Insgesamt wurden 22 Originalarbeiten im Hinblick auf die Definition von Rebound pain, die Annahme der Entstehung sowie Therapieoptionen ausgewertet. Dabei zeigt sich, dass keine einheitliche Definition existiert, die Pathophysiologie nicht eindeutig geklärt ist und zum aktuellen Zeitpunkt auch noch keine klaren Empfehlungen zu Prophylaxe sowie Therapie gegeben werden können.

**Zusatzmaterial online:**

Die Online-Version dieses Beitrags (10.1007/s00101-022-01120-z) enthält eine tabellarische Zusammenstellung über Studiendesign sowie Kernaussagen der vorgestellten Studien.

## Abstract (en)

**Background:**

Rebound pain as a side effect of regional anaesthesia is an excessive sensation of pain after the effect of local anaesthesia has subsided. This sensation goes well beyond the normal wound pain following a surgical intervention. This phenomenon has entered focus of research in the past 10 years now, but the specific causes are so far unclear and there are still no targeted treatment recommendations.

**Objective:**

This review article is intended to give the readership an overview of the current state of research about rebound pain. The theories of pathophysiology are presented and prophylaxis as well as treatment strategies are explained.

**Material and methods:**

For this review article, the publications about rebound pain that appeared from 2005 up to May 2021 in PubMed were reviewed and the authors’ definitions of rebound pain as well as the assumptions on pathophysiology and treatment recommendations were summarized.

**Results and discussion:**

A total of 22 original papers from the years 2005–2021 were evaluated regarding the differences between the definitions of rebound pain, the assumption of its occurrence as well as possible treatment options. It turns out that there is no uniform definition by the professional societies, the pathophysiology has not yet been clearly identified and no clear recommendations for prophylaxis or treatment can be given to date; however, early administration of pain medication (e.g. NSAIDs) before the end of the nerve block has proven to be helpful. Likewise, dexamethasone as an adjuvant to regional anaesthesia shows positive effects regarding the occurrence of rebound pain. In any case, it makes sense to provide patients with comprehensive information about this special side effect of regional anaesthesia so that those affected can correctly classify the excessive pain reaction. Targeted studies to avoid severe pain after regional anaesthesia, e.g. through the addition of adjuvants, are necessary in order to keep side effects as low as possible and thereby improve patient comfort and the acceptance of regional anaesthesia.

Das Auftreten von Rebound pain nach peripherer Nervenblockade ist ein Phänomen, welches in den letzten 10 Jahren stärker in den Fokus der anästhesiologischen Forschung geraten ist. Bisher ist jedoch noch unklar, ob es sich hierbei wirklich um ein eigenständiges Krankheitsbild handelt, welche Ursachen für die Entstehung mitverantwortlich sind und wie eine Prophylaxestrategie sowie mögliche zielgerichtete Therapien aussehen könnten. Dieser Übersichtsartikel soll den aktuellen Stand der Forschung zusammenfassen und der Leserschaft die Thematik des Phänomens darstellen.

## Falldarstellung

Eine gesunde 24-jährige Frau stellt sich nach einem Fahrradsturz um 10:30 Uhr in der unfallchirurgischen Notaufnahme mit schmerzender rechter Hand vor. In der durchgeführten Röntgendiagnostik zeigt sich eine dislozierte Mittelhandfraktur der Metacarpalia 4 und 5. Die Patientin wird zur chirurgischen Versorgung der Fraktur stationär aufgenommen und noch am gleichen Tag einer operativen Versorgung zugeführt. Nach der anästhesiologischen Aufklärung entscheidet sich die junge Frau für eine axilläre Blockade des Plexus brachialis in Kombination mit einer milden Sedierung. Sie wiegt 60 kg bei einer Größe von 1,75 m, nimmt außer eines oralen Kontrazeptivums keine Medikamente ein und wird somit als ASA I klassifiziert. Nach Durchführung der präoperativen Untersuchungen und Abwarten bis zur Nüchternheit wird die Patientin gegen 16:00 Uhr in den OP-Bereich eingeschleust. Die diensthabende Anästhesistin nimmt die junge Frau entgegen und lagert nach Klärung aller Formalitäten den Arm zur Anlage der axillären Plexusblockade. Bei der sportlichen Frau ist die Anatomie des Plexus brachialis in der Sonographie gut darstellbar; die additive elektrische Stimulation zeigt eine gute Reizantwort aller Zielnerven. Die Betäubung des Nervengeflechtes erfolgt mit insgesamt 30 ml Lokalanästhetikum (Mischung aus 10 ml Ropivacain, 0,2 %, und 20 ml Prilocain, 1 %). Um 16:20 Uhr erfolgt die Freigabe für die Operation, und die Patientin wird durch die Operateure gelagert und die Hand desinfizierend abgewaschen. Zur Sedierung erhält die Patientin einen 50 mg Bolus Propofol und anschließend 100 mg/h per continuitatem i.v. Die Testung der Schmerzempfindung vor Hautschnitt ist negativ und die Kollegen der Unfallchirurgie beginnen um 16:45 Uhr mit der Operation. Die komplikationslose Plattenosteosynthese der beiden Mittelhandknochen dauert insgesamt 1 h und 15 min, sodass die Patientin um kurz nach 18:00 Uhr aus dem OP ausgeschleust und zur kurzen postoperativen Überwachung in den Aufwachraum verlegt werden kann. Nach 30 min erfolgt die Verlegung auf die chirurgische Normalstation.

Um 22:30 Uhr wird der diensthabende Anästhesist von der unfallchirurgischen Kollegin angerufen, da es Probleme bei der jungen Patientin gibt. Der Facharzt für Anästhesie begibt sich umgehend auf die Station zur Patientin und findet diese mit stärksten Schmerzen im Bett sitzend vor. Die chirurgische Kollegin berichtet, dass sie gegen 22 Uhr von dem diensthabenden Krankenpfleger angerufen wurde, da die Patientin zunehmende Schmerzen im Arm angegeben hat. Die von dem Pfleger bereits gegen 21 Uhr veranlasste Gabe der Bedarfsmedikation (600 mg Ibuprofen p.o. und 1 g Metamizol als Kurzinfusion) habe zu keinerlei Besserung geführt. Die Chirurgin ordnete 7,5 mg Piritramid als Kurzinfusion an, entfernte die Verbände und untersuchte die Patientin grob orientierend neurologisch. Sie konnte aber keinen Hinweis auf eine operative Komplikation erkennen, woraufhin sie dann den anästhesiologischen Dienstarzt informierte. Der Anästhesist erhebt bei seiner Anamnese Folgendes:

- Schmerzintensität: 8–9/10 auf der numerischen Rating-Skala (NRS),
- Schmerzlokalisation: im Seitenvergleich von der Achsel abwärts den gesamten rechten Arm betreffend,
- Schmerzcharakter: heller Schmerz mit einem brennenden Gefühl, Hyperästhesie und Allodynie. Der Schmerz hat sehr plötzlich angefangen, wovon die Patientin wach geworden ist.

Auf Anordnung erhält die Patientin nochmals 7,5 mg Piritramid als Kurzinfusion. Hiernach kommt es zu keiner Besserung, sodass der Anästhesist repetitiv Morphinboli i.v. verabreicht. Nach insgesamt 10 mg Morphin gibt die Patientin die Schmerzen mit 5/10 an. Die Patientin erhält aufgrund des neuropathischen Schmerzcharakters und zum Schlafanstoß noch 300 mg Gabapentin p.o. Hiernach schläft sie bis 5 Uhr und meldet sich dann erneut wegen Schmerzen. Nun gibt sie jedoch einen auf die Hand betonten Schmerz der Intensität 6/10 an, der eher pochend beschrieben wird und nach 1 g Metamizol und 5 mg Piritramid schnell rückläufig ist.

Eine neurologische Untersuchung am nächsten Tag ergibt keinen Hinweis auf eine Nervenschädigung, und die Patientin kann nach Hause entlassen werden. Außer dem normalen Wundschmerz zeigt die Patientin keine neuerlichen Schmerzsensationen wie in der ersten postoperativen Nacht. Der weitere Verlauf und die Wundheilung sind unauffällig.

## Einleitung

Regionalanästhesieverfahren sind aus der modernen Anästhesie nicht mehr wegdenkbar. Durch die gezielte lokale Schmerzausschaltung werden perioperativ deutlich weniger Opioide eingesetzt, die Verfahren sind im Gegensatz zur Allgemeinanästhesie kaum kreislaufbelastend und auch wesentlich umweltschonender, die Patienten sind nach der Operation schneller erholt und können dadurch auch früher mobilisiert und entlassen werden [3, 5, 18]. Deshalb eignen sich periphere Nervenblockaden insbesondere für ambulante Operationen sowie auch für multimorbide Patienten [14]. Die Problematik des sog. Rebound pain ist jedoch bei der Anwendung von Regionalanästhesie in den letzten 10 Jahren vermehrt in den Fokus der anästhesiologischen Forschung geraten. Es handelt sich hierbei um ein übermäßig starkes Schmerzempfinden nach einer Operation (zumeist an einer Extremität), welche unter einer peripheren Nervenblockade (PNB) durchgeführt wurde [8]. Bisher existiert jedoch noch keine einheitliche Definition, mit welcher dieses Phänomen eingeordnet werden kann, und es bestehen lediglich Annahmen über die Entstehung der überschießenden Schmerzempfindung sowie uneinheitliche Therapieansätze.

## Methoden

Es wurde eine systematische Literaturrecherche von Studien, Metaanalysen, Übersichtsarbeiten und Reviews, in deutscher und englischer Sprache, veröffentlicht im Zeitraum von 2005–2021, unter Nutzung von PubMed durchgeführt. Es wurden 298 Suchergebnisse gefunden. Nach Ausschluss von Duplikaten und einem Titel‑/Abstract-Screening wurden 22 Manuskripte für diesen Übersichtsartikel berücksichtigt, wovon 9 im Detail näher dargestellt werden. Folgende Stichworte wurden verwendet: „rebound pain“, „rebound pain“ AND „regional anaesthesia“, „rebound hyperalgesia“, „rebound pain single shot block“, „peripheral nerve block“ AND „adjuvants“, „perineural dexamethasone“, „rebound pain“ AND „orthopedic surgery“.

## Definitionen

Auch wenn gegenwärtig keine einheitliche Definition für das Phänomen Rebound pain existiert, überschneiden sich die Darstellungen in den sich damit befassenden Studien (Tab. 1). Zur verständlichen Übersicht sind die Kernaussagen der jeweiligen Autoren zusammengefasst:

- Rebound pain wird als ein quantifizierbarer Unterschied der Schmerzwerte definiert, zu dem Zeitpunkt einer noch wirksamen peripheren Nervenblockade und dem Abklingen der Blockade [8, 11, 12, 14, 15, 21, 22]. Galos et al. (2016) schildern Rebound pain als eine schlecht beschriebene Entität, die allgemein als dramatische Zunahme der Schmerzen definiert wird, sobald die Regionalanästhesie nach einmaliger Injektion abgeklungen ist [4].
- Die beschriebenen Schmerzen nach Abklingen der Nervenblockade werden i. Allg. als mechanisch-chirurgischer Schmerz auf einen ungehinderten nozizeptiven Reiz präzisiert [11, 12].
- Dada et al. (2019) legen Rebound pain als einen Zustand der Hyperalgesie in einem Zeitraum von 8 bis 24 h nach Anlage einer peripheren Nervenblockade fest [2].
- Des Weiteren charakterisieren Barry et al. (2020) Rebound pain als einen Übergang von gut kontrollierten Schmerzen auf der numerischen Rating-Skala (NRS) ≤ 3 zu einem Schmerzniveau von NRS ≥ 7. Barry et al. beziehen sich dabei auf einen Zeitraum von 24 h nach Anlage der peripheren Nervenblockade [1]. Diese Definitionen streben mit der Festlegung von NRS-Werten eine präzisere Qualifizierung sowie Quantifizierung des Rebound pain an.
- Nehmen die meisten Autoren nur Bezug auf die wahrgenommenen, quantifizierbaren Schmerzen, erweitern Muñoz-Leyva et al. (2020) in ihrer Definition Rebound pain auch auf die Auswirkungen des psychischen Wohlbefindens, die Qualität der Erholung sowie die Aktivitäten im Alltag [14].

**Table 1:** 

Autor	Jahr	Definition (deutsche Übersetzung)	Definition (englisches Original)
Williams et al. [21]	2007	„Quantifizierbarer Unterschied in den Schmerzwerten bei wirksamer Blockade gegenüber der Zunahme der akuten Schmerzen in den ersten Stunden nach Abklingen der Effekte einer perineuralen Einzelinjektion oder einer kontinuierlichen Infusion von Lokalanästhetika“	„Quantifiable difference in pain scores when the block is working versus the increase in acute pain encountered during the first few hours after the effects of peri-neural single-injection or continuous infusion local anesthetics resolve“
Williams [22]	2012		
Kolarczyk und Williams [11]	2011	„Rebound-Schmerzen nach dem Abklingen einer peripheren Nervenblockade werden i. Allg. als mechanisch-chirurgische Schmerzen angesehen, die aus der Beendigung einer Nervenblockade mit ungehindertem nozizeptivem Reiz resultieren“	„Rebound pain after a peripheral nerve block resolves is generally considered to be comprised of the mechanical-surgical pain that results from the resolution of a nerve block with unopposed nociceptive input“
Galos et al. [4]	2016	„Eine schlecht beschriebene Entität, die allgemein als dramatische Zunahme der Schmerzen definiert wird, sobald die Regionalanästhesie nach einmaliger Injektion abgeklungen ist“	„A poorly described entity that is commonly defined as a dramatic increase in pain once single-injection regional anesthesia has dissipated“
Lavand’homme [12]	2018	„Ein mechanisch-chirurgischer Schmerz bei einem ungehinderten nozizeptiven Reiz, nach Abklingen der peripheren Nervenblockade“	„A mechanical-surgical pain from unopposed nociceptive input that is uncovered when PNB wears off“
Dada et al. [2]	2019	„Zustand einer Hyperalgesie im Zeitraum von 8 bis 24 h nach Anlage der peripheren Nervenblockade“	„State of hyperalgesia with an onset between 8 and 24 h after block administration“
Nobre et al. [15]	2019	„Rebound pain ist definiert als der quantifizierbare Unterschied der Schmerzwerte, wenn eine periphere Nervenblockade wirkt, im Vergleich zu den akuten Schmerzen, die festgestellt werden, wenn die Blockade abklingt“	„Rebound-Pain is defined as the quantifiable difference in pain scores when a PNB is working versus the acute pain found when the blockade stops working“
Muñoz-Leyva et al. [14]	2020	„Wesentliche Merkmale von Rebound pain sind, dass es sich um akute postoperative Schmerzen handelt, welche nach Abklingen der PNB auftreten und klinisch signifikant sind, entweder in Bezug auf die Schmerzintensität, die Auswirkungen auf das psychische Wohlbefinden, die Qualität der Erholung und die Aktivitäten im Alltag“	„Essential characteristics of Rebound-Pain are that it is acute postoperative pain, ensues following resolution of PNB, and is clinically significant, either with regard to the intensity of pain or the impact on psychological well-being, quality of recovery, and activities of daily living“
Barry, Garrett S. et al. [1]	2020	„Ein Übergang von einem gut kontrollierten Schmerz-NRS ≤ 3, während die Nervenblockade wirkt, zu einem starken Schmerz NRS ≥ 7 innerhalb von 24 h nach Anlage der Nervenblockade“	„A transition from well-controlled pain NRS ≤ 3 while the block is working to severe pain NRS ≥ 7 within 24 h of block performance“

Laut aktueller Studienlage ist Rebound pain somit vom einsetzenden Wundschmerz nach Abklingen der Lokalanästhetikawirkung nach erfolgter Regionalanästhesie abzugrenzen. Der lokale Wundschmerz ruft – insbesondere wenn keine Basisanalgesie vor Abklingen der Blockade verabreicht wurde – gelegentlich ebenfalls heftige Schmerzen hervor, ist aber letztlich durch Opioidgabe rasch beherrschbar. Klare Unterscheidungskriterien zum Rebound pain sind aber bisher nicht eindeutig definiert.

## Pathomechanismus

Weshalb es bei manchen Patienten zu einer überschießenden Schmerzempfindung nach Abklingen der Regionalanästhesie im Sinne von Rebound pain kommt, ist noch nicht vollständig geklärt. Eine überschießende Schmerzempfindung bedeutet, dass der jeweilige Schmerz über dem gewohnten Niveau liegt. Dies ist sowohl subjektiv durch eine vom Patienten bewertete Schmerzskala und seine Einschätzung zu gewohnten Schmerzen messbar als auch objektiv im Vergleich zu der Gesamtheit der Patienten mit gleichem Beschwerdebild. Es gibt jedoch schon einige Untersuchungen, die den Pathomechanismus für das Auftreten von Rebound pain zu ergründen versuchen. Chirurgische Verletzungen, v. a. solche, die eine osteosynthetische Versorgung der oberen Extremitäten benötigen, stellen neben dem Alter, dem Geschlecht, starken präoperativen Schmerzen und dem fehlenden perioperativen Gebrauch von Dexamethason einen signifikanten Risikofaktor für das Auftreten von Rebound pain dar [1, 2]. Hierbei ist zu erwähnen, dass bis auf den fehlenden Gebrauch von Dexamethason alle genannten Risikofaktoren generell Prädiktoren für postoperative Schmerzen sind. Die daraus resultierende Sensibilisierung sowohl im peripheren als auch im zentralen neuronalen Bereich führt vermutlich zu einer verstärkten und kontinuierlichen Signalantwort aus den peripheren Nozizeptoren. Diese gesteigerte Schmerzsensibilisierung kann zu Hyperalgesie und Allodynie führen. Zudem wird vermutet, dass die in diesem Prozess entstandene Sensibilisierung bei Beendigung einer PNB eine verstärkte Schmerzempfindung provoziert [2]. In einer Untersuchung von Kolarczyk und Williams konnte gezeigt werden, dass Ratten, die eine PNB mit Ropivacain, 0,5 %, erhielten, im Vergleich zur Kontrollgruppe 3 h nach der Injektion eine Hyperalgesie nach Hitzestimulation entwickelten [11]. Muñoz et al. geben stattdessen an, dass eine Hyperalgesie als eine normale Konsequenz betrachtet werden muss, die sowohl im Operationsgebiet als auch im angrenzenden gesunden Gewebe im Sinne einer physiologischen Reaktion stattfindet. Des Weiteren erläutern die Autoren, dass eine PNB keinen signifikanten Einfluss auf das periphere Gebiet um die Verletzung habe. Die physiologische Entzündungsreaktion läuft ohne systemisch wirksame Medikation ungehindert ab, mit dem Ergebnis, dass der hyperalgetische Bereich nach dem Abklingen der PNB verstärkt nozizeptiv aktiv ist [14]. Der Zusammenhang zwischen der Applikation von Lokalanästhetika und dem Auftreten von Rebound pain wird ebenso kontrovers gesehen. Durch ihre reversible Wirkung an spannungsabhängigen Natriumkanälen wirken sie primär analgetisch durch eine Blockade der axonalen Transduktion, jedoch wird zusätzlich eine antiinflammatorische Wirkung durch systemische Resorption diskutiert. Eine Studie von Martin et al. über Lidocain zeigte einen systemisch wirksamen antiinflammatorischen Effekt bei chirurgischen Interventionen, wohingegen Gordon et al. darstellten, dass es bei dentalen Eingriffen unter Applikation von Bupivacain zu einer gesteigerten Expression von Zyklooxygenase‑2 (COX-2) kommt, was wiederum zu einer erhöhten Prostaglandin(PGE_2_)-Synthese führt und somit eine stärkere Schmerzempfindung zur Konsequenz hat [2, 7, 13]. Andere Studien zeigen bei der Anwendung von Bupivacain eine übermäßige Produktion von reaktiven Sauerstoffspezies sowie eine gesteigerte Autophagie der Zellen [2]. Des Weiteren beschreiben Dada et al. die Gemeinsamkeiten von Rebound pain und Schmerzcharakteren wie Allodynie und Hyperalgesie mit einer gesteigerten Erregbarkeit der Nozizeptoren und damit verbundener vermehrter Aktivierung von schmerzassoziierten C‑Fasern, ohne dass eine Nervenläsion in mechanischer Form stattgefunden hat [2, 12]. Sie legen auch dar, dass für solche Schmerzentitäten eine gesteigerte Aktivierung von mechanoinsensitiven Nozizeptoren, „silent nociceptors“, stattfindet [2]. Als weiterer möglicher Pathomechanismus wird das Auftreten von ischämisch bedingten Nervenläsionen als Folge einer PNB diskutiert. Durch eine Verletzung oder einen Verschluss der epineuralen Gefäße kann es zu einer Minderversorgung des zu versorgenden Nervenabschnitts kommen, was wiederum zu einer Irritation desselbigen führt. Eine Ischämie der epineuralen Gefäße kann durch verschiedene Ursachen begründet sein, z. B. durch direkten Verschluss oder Schaden, wie z. B. durch Anlage einer pneumatischen Sperre bei blutleeren Operationen, durch perineurale Blutungen, intrafaszikuläre Injektionen oder „High-pressure“-Injektionen [2]. Die oftmals postulierte nervale Ischämie durch Adrenalinzusatz zum Lokalanästhetikum ist vermutlich eher von minderer Relevanz [19].

## Möglichkeiten der Prophylaxe und Therapie

Aufgrund der noch nicht ausreichenden Erforschung des Phänomens Rebound pain und den damit im Zusammenhang stehenden Vorgängen bestehth eine mögliche Zielsetzung in der Vermeidung dieses Phänomens durch geeignete Prävention, bevor es einer Therapie bedarf. Die Empfehlungen der sich mit dieser Thematik befassenden Autoren werden hier vorgestellt.

Im Allgemeinen könnte sich die geeignete Prävention und Therapie aus diversen Bausteinen zusammensetzen. Hierzu zählen nach Muñoz-Leyva et al. neben der angemessenen Aufklärung des Patienten, ein multifaktoriell gewählter Ansatz des genutzten Analgesieverfahrens sowie die frühzeitige Einnahme von Analgetika vor Abklingen der Nervenblockade. Dieser Ansatz kann beispielsweise durch die Nutzung von kontinuierlichen Kathetertechniken oder Adjuvanzien ergänzt werden, um eine Verlängerung der Wirkdauer herbeizuführen [14].

### Aufklärung und Beratung der Patienten

Eine Prävention sollte stets mit der individuellen Aufklärung der Patienten über die möglichen Komplikationen einer peripheren Nervenblockade erfolgen, damit die Patienten eine angemessene Erwartung in Bezug auf das Abklingen der Nervenblockade entwickeln können. Hierbei kann auf den Aspekt der überschießenden Schmerzreaktion eingegangen werden, wobei jedoch deutlich gemacht werden sollte, dass dieses Phänomen noch nicht abschließend geklärt ist. Des Weiteren kann durch die Aufklärung der Patienten eine erhöhte Compliance zur Einnahme von fest verordneten Analgetika im noch schmerzfreien Zustand angestrebt werden [6, 14]. Goldstein et al. präzisieren die Einnahme von oralen Analgetika auf den Zeitraum von etwa einer bis 2 h vor dem Abklingen der Nervenblockade. Die Wirkdauer der PNB wird hierbei basierend auf den genutzten Lokalanästhetika abgeschätzt [6].

### Verlängerung der sensorischen Blockade

Einen möglicherweise weiteren Ansatz zur Verhinderung von Rebound pain stellt die Nutzung einer kontinuierlichen Nervenblockade durch einen Schmerzkatheter dar. Dieser wird eingesetzt, um die sensorische Blockade zu verlängern, sowie durch Ausschleichen der Medikation ein abruptes Ende der Schmerzblockade zu umgehen. Die Verlängerung der Schmerzblockade generiert ebenso Zeit, um dem Körper die Möglichkeit zu geben, den durch die Operation entstandenen Entzündungsprozess zu heilen oder abklingen zu lassen. Um eine deutliche Verminderung von Rebound pain zu erreichen, bedarf es einer signifikanten Verlängerung der Analgesiezeit, verglichen mit der Wirkdauer der einzeitigen PNB. Ebenso muss die postoperative Zeit mit dem Patienten geplant werden, da es zu einer längeren Phase der sensomotorischen Einschränkung kommt. Dieser Ansatz eignet sich somit nur für den stationären Bereich und kann im ambulanten Setting kaum real genutzt werden [6, 14].

### Adjuvanzien bei Injektion einer einzeitigen Nervenblockade

Zur Verlängerung der Wirksamkeit der peripheren Nervenblockade bietet sich neben der Nutzung von katheterbasierten Verfahren auch die zusätzliche Gabe von Adjuvanzien während der Anlage der „Single-shot“-Anästhesie an [14].

Dexamethason gilt, systemisch oder perineural appliziert, als wirksames Adjuvans, um eine Verlängerung der sensorischen Nervenblockade herbeizuführen [16, 20]. Neben der Verlängerung der Wirkdauer des genutzten Lokalanästhetikums hat Dexamethason weitere positive Effekte für den Patienten. Es führt zu einer Reduktion des Opioidkonsums in den ersten 24 h postoperativ, es verringert die beschriebene Schmerzintensität am Punkt der stärksten Schmerzempfindung postoperativ und begünstigt eine bessere Schlafqualität in der Nacht nach der Operation [3]. Bei ggf. notwendiger postoperativer Opioidgabe verringert es darüber hinaus auch das Risiko von opioidbedingter Nausea und Emesis [5]. Wiesmann et al. zeigen in ihrem Übersichtsartikel auf, dass es weiterer Forschung bedarf, ob eine perineurale Applikation von Dexamethason der zentralen Gabe zu bevorzugen ist [20]. Hingegen raten Tan et al. von einer routinemäßigen perineuralen Gabe von Dexamethason ab und präferieren die systemische Gabe [17].

Neben der Gabe von Dexamethason in Kombination mit einem Lokalanästhetikum wurden ebenso weitere Adjuvanzien, u. a. Dexmedetomidin, Buprenorphin, Clonidin, Midazolam und Epinephrin kombiniert [9, 10]. Bei der Anwendung dieser Adjuvanzien konnte eine Reduzierung der Gesamtdosis der Lokalanästhetika erreicht und die Wirkdauer der Anästhesie verlängert werden [10]. Nach Kirksey et al. scheint es, als seien folgende Adjuvanzien gut geeignet für eine koinzidierende Verlängerung der Nervenblockade: Buprenorphin, Clonidin, Dexamethason, Dexmedetomidin und Magnesium [9]. Jedoch werden ebenso Bedenken geäußert, da neurale Nebenwirkungen sowie eine fragliche Toxizität der Adjuvanzien bislang nicht ausreichend untersucht wurden [9]. Knight et al. stehen dem gegenüber und beschreiben eine Verbesserung der Analgesie bei Verwendung von Dexmedetomidin als Adjuvans, ohne eine zeitgleiche Risikoerhöhung der Neurotoxizität von Lokalanästhetika beobachtet zu haben [10].

Ob das Phänomen Rebound pain tatsächlich durch die Gabe von Dexamethason oder weiterer Adjuvanzien reduziert werden kann, muss noch weitergehend untersucht werden. Erste Daten zeigen allerdings, dass Patientengruppen ohne Dexamethasongabe signifikant häufiger Rebound pain angegeben haben als Patientengruppen, die Dexamethason perioperativ erhielten [3].

### Multifaktorielle Schmerztherapie

Bei einer peripheren Nervenblockade kommt es ausschließlich zur Blockade der nozizeptiven Schmerzweiterleitung vom Ort des Reizes zum Rückenmark und somit zu keiner Schmerzwahrnehmung [14].

Um auch weitere Ursachen der Schmerzentstehung in die Analgesie miteinzubeziehen, benötigt man ein multimodales (multifaktorielles) Analgesiekonzept [2, 14, 15]. Dieses sollte insbesondere zur Abschwächung der folgenden pathophysiologischen Faktoren führen:

1. nozizeptive Reize,
2. Prozesse peripherer Sensibilisierung,
3. humorale Entzündungsreaktionen.

Ein multifaktorielles Analgesiekonzept besteht in diesem Kontext aus beispielsweise Metamizol (bevorzugt verwendet im deutschsprachigen Raum) oder Paracetamol (zumeist verwendet im angloamerikanischen Bereich), nichtsteroidalen Antirheumatika bzw. COX2-Hemmern und oralen Opioiden [14].

Zum aktuellen Stand der Wissenschaft existiert kein Nachweis, dass ein multifaktorielles Analgesiekonzept Rebound pain verhindern kann. Nichtsdestotrotz sollte ein umfassendes Analgesiekonzept routinemäßig im Rahmen einer hochwertigen klinischen Praxis verordnet werden [14].

## Bewertung der aktuellen Studienlage

Rebound pain ist bisher kein klar definiertes Krankheitsbild, sondern vielmehr eine Ausschlussdiagnose, wenn andere Ursachen als Erklärung für die überschießende Schmerzreaktion nicht in Betracht gezogen werden können. Da viele Faktoren auf die Schmerzentstehung einwirken, ist ein kausaler Zusammenhang nicht immer eindeutig gegeben. Mit diesem Übersichtsartikel wird der aktuelle Stand der Forschung vorgestellt, und es soll insbesondere auf die Begriffsproblematik aufmerksam gemacht werden. Man erkennt bei dem Vergleich der einzelnen Definitionen (Tab. 1 und Zusatzmaterial online), dass jeder Autor eine etwas differierende Ansicht über Rebound pain hat. Nur durch eine eindeutige Definition von Rebound pain können eine bessere Vergleichbarkeit aktueller Forschungsergebnisse sowie spezifischere Forschung zu diesem Phänomen ermöglicht werden. Gezielte Untersuchungen zur Vermeidung von starken Schmerzen nach Regionalanästhesie, z. B. durch den Zusatz von Adjuvanzien, sind erforderlich, um Nebenwirkungen möglichst gering zu halten und dadurch den Patientenkomfort und die Akzeptanz von Regionalanästhesie zu verbessern. Dem aufmerksamen Leser wird nicht entgangen sein, dass die bisher vorliegenden Studienergebnisse hierzu teilweise widersprüchlich sind und dementsprechend in Zukunft weiter spezifiziert werden sollten.

## Zusammenfassung

Auch wenn das Phänomen nicht alle Patienten nach einer Regionalanästhesie gleichermaßen betrifft, kann es individuell sehr ausgeprägt auftreten und für den Betroffenen sehr belastend sein. Möglicherweise wird durch das Auftreten von Rebound pain der initial opioidsparende Effekt der Regionalanästhesie teilweise relativiert. Nach aktuellem Forschungsstand erscheint es zwar so, dass Rebound pain nur zu einer vorübergehenden Beeinträchtigung führt und keine langfristigen Nervenschäden oder chronifizierende Schmerzsyndrome dadurch hervorgerufen werden, nichtsdestotrotz erzeugt der überschießende Schmerz postoperativ einen erhöhten Opioidbedarf und vermindertes Wohlbefinden der Patienten und führt zu einer längeren Regenerationsphase [14]. In Bezug auf die optimale Therapie besteht weiterhin Unklarheit. Es erscheint hilfreich, die Patienten über die möglicherweise entstehende Schmerzsensation umfassend aufzuklären und Hilfestellungen (z. B. frühzeitige, überlappende Einnahme einer Schmerzmedikation zum Ende der Nervenblockade) an die Hand zu geben. Insbesondere ambulante Patienten benötigen einen Ansprechpartner, an den sie sich wenden können, sofern es zu Problemen im häuslichen Umfeld kommt [14]. Erste Versuche einer perineuralen Applikation von Dexamethason erscheinen vielversprechend [3].

Der oben dargestellte Vergleich der aktuellen Literatur zum Thema Rebound pain zeigt jedoch auch, dass eine klare Definition des Phänomens weiterhin aussteht. Klinische und experimentelle Studien zu Risikofaktoren, Prophylaxe- und Therapieoptionen sind dringend nötig, um ein solches „big little problem“ der postoperativen Phase besser zu verstehen und vermeidbar zu machen.

## Fazit für die Praxis

- Rebound pain wird in der aktuellen Literatur als Nebenwirkung der Regionalanästhesie beschrieben, welche als überschießende Schmerzreaktion nach Beendigung der sensorischen Blockade auftritt.
- Der initial opioidsparende Effekt der Regionalanästhesie wird durch dieses Phänomen aufgehoben.
- Es existiert bisher keine einheitliche Definition innerhalb der Fachgesellschaften, wodurch eine zielgerichtete Forschung erschwert wird.
- Da die Pathophysiologie nicht eindeutig geklärt ist, können auch noch keine definitiven Empfehlungen zu Prophylaxe sowie Therapie gegeben werden.
- Patienten sollten umfassend aufgeklärt werden, damit sie mögliche überschießende Schmerzen nach Abklingen der Blockade einordnen können.
- Eine frühzeitige Gabe von Schmerzmedikamenten (z. B. NSAR) vor Beendigung der Nervenblockade hat sich jedoch als hilfreich erwiesen. Ebenso zeigt Dexamethason als Adjuvans zur Regionalanästhesie positive Effekte gegenüber dem Auftreten von Rebound pain.

## Supplementary Information



