# Supplementary material for: Rebound pain—From definition to treatment
Source: Anaesthesiologie. 2022 May 5;71(8):638–45. [Article in German] doi: 10.1007/s00101-022-01120-z (PMC9352600; doi:10.1007/s00101-022-01120-z)
Supplement: Supplementary file 1 [file 101_2022_1120_MOESM1_ESM.pdf]

Zusatzmaterial zum Beitrag **„Rebound Pain – Von der Definition bis zur Therapie" von Streb T, Schneider A, Wiesmann T et al. (2022) in *Der Anaesthesist.***
Beitrag und Zusatzmaterial stehen Ihnen auf www.springermedizin.de zur Verfügung. Bitte geben Sie dort den Beitragstitel in die Suche ein.

Übersicht über Studiendesign sowie die Kernaussagen der vorgestellten Studien

Autor	Jahr	Definition (deutsche Übersetzung)	Definition (englisches Original)	Studientyp	Methode	Einschlüsse	Ergebnis / Conclusion
Williams et al. [21]<br><br>Williams [22]	2007<br><br>2012	„Quantifizierbarer Unterschied in den Schmerzwerten bei wirksamer Blockade gegenüber der Zunahme der akuten Schmerzen in den ersten Stunden nach Abklingen der Effekte einer perineuralen Einzelinjektion oder einer kontinuierlichen Infusion von Lokalanästhetika."	„Quantifiable difference in pain scores when the block is working versus the increase in acute pain encountered during the first few hours after the effects of peri-neural single-injection or continuous infusion local anesthetics resolve."	Randomisiert kontrollierte Studie	Nach standardisierter Spinalanästhesie und perioperativer multimodaler Analgesie erhielten die Patienten einen femoralen Perineuralkatheter und 50 Stunden entweder Placebo oder Levobupivacain. Alle Patienten erhielten vor der Infusion Levobupivacain (30 ml 0,25 % als Bolus). Die Patienten führten 6 Tage lang ein Schmerztagebuch, das fortlaufende NRS-Scores und die Wahrnehmung über das Ende der Blockade. Blockdauer und Rebound-Pain-Scores wurden berechnet.	Es wurden die Daten von 106 Patienten erhoben, wobei von nur 84 Patienten die Schmerztagebücher hinreichend waren um die Dauer der Nervenblockade und den „RPS Rebound pain score"zu bestimmen.	Patienten, die eine Infusion mit Placebo erhielten, berichteten über eine mittlere Dauer der Nervenblockade von 37 Stunden gegenüber 59 Stunden bei Patienten, die eine Levobupivacain-Infusion erhielten (P < 0,001). Die mittleren Rebound-Pain-Scores stiegen um 2,0 (95 % KI: 1,6, 2,4). Basierend auf den Berechnungen, die zum Ableiten von Blockdauer- und Rebound-Pain-Scores verwendet wurden, war jede Stunde zusätzlicher Blockdauer prädiktiv für eine Verringerung der Rebound-Pain-Scores um 0,03 Einheiten.
Kolarczyk and Williams [11]	2011	„Rebound Schmerzen nach dem Abklingen einer peripheren Nervenblockade werden im Allgemeinen als mechanisch-chirurgische Schmerzen angesehen, die aus der Beendigung einer Nervenblockade mit ungehindertem	"Rebound-Pain after a peripheral nerve block resolves is generally considered to be comprised of the mechanical-surgical pain that results from the resolution of a nerve	Randomisiert kontrollierte Tierversuchst udie	Unter chirurgischer Exposition wurde eine Blockade des linken N. ischiadicus bei männlichen Ratten durchgeführt. Die Wirksamkeit und Dauer der Blockierung wurde mit seriellen Hitze-, mechanischen und taktilen Tests bewertet; Motorpropriozeptive- und Sedierungstests wurden 1 h und 7 h nach der Injektion	An 2 Gruppen erwachsener männlicher Ratten durchgeführte Testung– Ropivacain (200 µl, 0,5%, n=14) versus Kochsalzlösung (n=11).	Vollständige durch Ropivacain induzierte Anästhesie nach 1 Stunde. 3 Stunden nach der Injektion war eine Hitzeüberempfindlichkeit im Rahmen einer abgeklungenen mechanischen Analgesie vorhanden. Alle Verhaltensmessungen kehrten 2 Wochen nach der Injektion auf den Ausgangswert zurück. 12–14 Tage nach der Injektion gab es keine Hinweise auf (i) Verhaltenssedierung, (ii) anhaltende

		nozizeptivem Reiz resultieren.“	block with unopposed nociceptive input.”		durchgeführt. Das Vorliegen einer Nervenverletzung wurde durch Wiederholung der Wärme-, Tast- und Motoriktests 12–14 Tage nach der Injektion beurteilt.		Veränderungen der Hitze- oder mechanischen Empfindlichkeit oder (iii) anhaltende Veränderungen der propriozeptiv-motorischen Funktion.<br><br>Ropivacain 0,5 % induziert vorübergehende Hitze-Hyperalgesie im Rahmen einer abgeklungenen mechanischen Analgesie, was weiter auf Modalität und/oder nozizeptive Faserspezifität hindeutet. Ob dieser Befund teilweise zu „Rebound-Pain“ führt, nachdem die Nervenblockaden der Patienten nachgelassen haben, muss weiter untersucht werden.
Galos et al. [4]	2016	„Eine schlecht beschriebene Entität, die allgemein als dramatische Zunahme der Schmerzen definiert wird, sobald die Regionalanästhesie nach einmaliger Injektion abgeklungen ist.“	“A poorly described entity that is commonly defined as a dramatic increase in pain once single-injection regional anesthesia has dissipated.”	Multizentrische Randomisiert kontrollierte Studie	Von den 36 eingeschlossenen Patienten wurden 18 in jede Gruppe randomisiert: Vollnarkose oder Plexus-Brachialis-Blockade. Die im Aufwachraum verabreichten Medikamente wurden erfasst. Die Patienten wurden entlassen und erhielten Oxycodon und Paracetamol 5/325 mg zur Schmerzkontrolle, und VAS-Formulare wurden bereitgestellt. Die Patienten wurden in festgelegten Intervallen postoperativ (2h, 4h, 6h, 12h, 24h, 48h und 72h) angerufen, um unter Verwendung der VAS Schmerzwerte zu sammeln und die Dosen der eingenommenen Analgetika zu dokumentieren. Darüber hinaus hatten die Patienten regelmäßige Nachuntersuchungen nach 2, 6 und 12 Wochen. Bei diesen Besuchen wurden erneut	40 Einschlüsse<br><br>36 ausgewertete Teilnehmer<br><br>4 lehnten Teilnahme ab<br><br>Keine Drop-outs	Patienten, die eine Vollnarkose erhielten, hatten 2h postoperativ schlechtere Schmerzwerte (Vollnarkose 6,7 ± 2,3 vs. Brachialplexusblockade 1,4 ± 2,3; mittlere Differenz 5,381; 95 % KI, 3,850–6,913; p < 0,001); wohingegen die berichteten Schmerzen nach 12h bei Patienten schlimmer waren, die eine Plexus-brachialis-Blockade erhielten (Vollnarkose 3,8 ± 1,9 vs.. Brachialplexusblockade 5,3 ± 2,5; mittlere Differenz -1,492; 95 % KI, -3,105 bis 0,120; p = 0,031). Es gab keinen Unterschied in der Operationszeit (Vollnarkose 119 ± 16 Minuten vs. Plexus-brachialis-Blockade 125 ± 23 Minuten; p = 0,432), aber die Zeit im Aufwachraum war länger bei Patienten, die eine Vollnarkose erhielten (284 ± 137 Minuten vs. 197 ± 90; p = 0,0398). Patienten, die eine Vollnarkose erhielten, nahmen mehr Fentanyl (64 µg ± 93 µg vs. 6,9 µg ± 14 µg; p < 0,001) und Morphin (2,9 µg ± 3,6 µg vs. 0,0 µg; p < 0,001) ein als

					Schmerzwerte unter Verwendung des VAS aufgezeichnet.		Patienten, die eine Brachialplexusblockade erhielten. Die funktionellen Ergebniswerte unterschieden sich nicht nach 6 Wochen oder 12 Wochen postoperativ.<br><br>Patienten, die eine Plexus-brachialis-Blockade erhielten, erlebten jedoch zwischen 12 und 24 Stunden nach der Operation eine Zunahme der Schmerzen. Die Anerkennung von „Rebound-Pain“ nach der Anwendung einer Regionalanästhesie in Verbindung mit einer Patientenberatung bezüglich einer frühzeitigen Narkotikaverabreichung kann den Patienten eine effektivere postoperative Schmerzkontrolle ermöglichen. Es ist wichtig, präoperativ mit den Patienten darüber zu sprechen, was sie in Bezug auf Rebound-Pain und postoperative Schmerzkontrolle zu erwarten haben, und ihnen zu raten, bei der Einnahme von Schmerzmitteln vor dem Abklingen der Regionalanästhesie aggressiv vorzugehen, um in ihrer Schmerzkontrolle immer einen Schritt voraus zu sein.
Lavand’homme [12]	2018	„Ein mechanisch-chirurgischer Schmerz bei einem ungehinderten nozizeptiven Reiz, nach Abklingen der peripheren Nervenblockade.“	“A mechanical – surgical pain from unopposed nociceptive input that is uncovered when PNB wears off “	Review			Die wichtigste Erkenntnis ist das Fehlen großer prospektiver Studien. Die Inzidenz von Rebound-Pain ist nicht bekannt, könnte aber 40 % der Patienten bei Auflösung der PNB erreichen. Pathophysiologische Mechanismen sind bis heute umstritten: mechanische und chemische (proinflammatorische Wirkung von Lokalanästhetika) Nervenschädigung durch PNB bei prädisponierten Patienten (mit starken präoperativen

							Schmerzen, jüngere Patienten). Auch fehlen wirksame Präventionsstrategien (z. B. Rolle analgetischer Adjuvantien bei PNB). Langfristige Folgen in Bezug auf die funktionelle Wiederherstellung und anhaltende Schmerzen wurden nicht nachgewiesen. Interviews mit Patienten haben die Notwendigkeit von Informationen und Aufklärung über PNB und postoperative Analgesie unterstrichen.
Dada et al. [2]	2019	„Zustand einer Hyperalgesie im Zeitraum von 8 bis 24 Stunden nach Anlage der peripheren Nervenblockade“	“State of hyperalgesia with an onset between 8 and 24 h after block administration.“	Review	Für diese Übersichtsarbeit wurde eine Suche nach Manuskripten durchgeführt, die zwischen Januar 2014 und Januar 2019 in englischer Sprache veröffentlicht wurden, und zwar mit PubMed, EMBASE und Web of Science.	Insgesamt wurden 768 Manuskripte identifiziert, von denen 408 Duplikate waren. Daher wurden 360 Artikel einem Titel-/Abstract-Screening unterzogen.<br><br>Es wurden insgesamt 28 Artikel in dieses Review eingeschlossen. Die Hälfte dieser Manuskripte betraf Patienten mit Rebound-Pain nach Blockaden der oberen Extremität, während die andere Hälfte Patienten mit Rebound-Pain nach Blockaden der unteren Extremität betraf.	Rebound-Pain nach peripheren Nervenblockaden für orthopädische Operationen können den Gesamtnutzen der Regionalanästhesie verringern. Multimodale Strategien wie präventive Opioid-Analgesie vor Abklingen der Blockade, die Anwendung intraartikulärer oder intravenöser steroidaler und nicht-steroidaler entzündungshemmender Medikamente, die Verwendung von Adjuvantien in Nervenblockaden zur Verlängerung der Analgesie und kontinuierliche Blockaden (gegenüber Single Shot Blockaden) können verwendet werden, um das Auftreten von Rebound-Pain zu reduzieren.<br><br>Ein optimaler Ansatz zur Reduzierung von Rebound-Pain bleibt jedoch ungewiss. Ein besseres Verständnis der Auswirkungen von Rebound-Pain auf die langfristige Schmerzkontrolle und den Opioidkonsum sowie die Entwicklung von Strategien zu ihrer Verhinderung werden erforderlich sein...

Nobre et al. [15]	2019	„Rebound Pain ist definiert als der quantifizierbare Unterschied der Schmerzwerte, wenn eine periphere Nervenblockade wirkt, im Vergleich zu den akuten Schmerzen, die festgestellt werden, wenn die Blockade abklingt.“	“Rebound-Pain is defined as the quantifiable difference in pain scores when a PNB is working versus the acute pain found when the blockade stops working.”	Review	Im Dezember 2017 wurde eine Literaturrecherche in der PubMed-Datenbank durchgeführt. Die verwendeten Suchbegriffe waren Rebound AND Pain AND Nerve Block, und es wurden 28 Ergebnisse erzielt. Die Einschränkung der Suche auf Artikel, die in den letzten fünf Jahren veröffentlicht wurden, ergab 22 Ergebnisse, die alle in englischer Sprache verfasst waren. Nach dem Lesen der Abstracts der 22 Artikel wurden 19 Artikel ausgewählt, die sich mit dem zu analysierenden Thema befassen. Zusätzlich zu den aus der Recherche ausgewählten Artikeln wurden auch 31 darin referenzierte Artikel konsultiert. Die Analyse umfasste 50 Artikel, die sich mit dem Auftreten des Rebound-Painphänomens bei verschiedenen Arten von Operationen befassten, deren Eingriffe sowohl im Krankenhaus als auch in der Ambulanz durchgeführt wurden.	19 Artikel aus der Literaturrecherche<br><br>Plus<br><br>31 referenzierte Artikel	Rebound-Pain ist eine Entität unbekannter Ätiologie, die die Qualität der postoperativen Genesung des Patienten beeinträchtigt. Die Beurteilung von Rebound-Pain sollte in der klinischen Praxis immer berücksichtigt werden, da es sich nicht um eine seltene Nebenwirkung von PNB handelt.<br><br>Zur Vorbeugung von Rebound-Pain empfehlen wir den Einsatz von PNB-Techniken, die Nervenschäden verhindern, und eine angemessene perioperative Analgesie in Verbindung mit der Patientenaufklärung über die frühzeitige Anwendung von Analgetika, selbst bei Vorhandensein einer durch PNB bereitgestellten Analgesie.<br><br>Trotz der Bemühungen, das Rebound-Painphänomen besser zu verstehen und Risikopatienten zu identifizieren, bleiben seine Behandlung und Prävention unangemessen.<br><br>Die Ergebnisse zu diesem Rebound-Pain-Phänomen in der aktuellen Literatur sind noch spärlich und die analysierten Studien zeigen mehrere Einschränkungen in Bezug auf Schmerz-Score-Messungen und Daten zu postoperativen Analgetika, was die Analyse der klinischen Implikationen dieses Ereignisses erschwert.<br><br>Umfangreiche prospektive Studien und weiterführende Studien zum besseren Verständnis der Pathophysiologie,

							Prävalenz, assoziierter Risikofaktoren und präventiver Methoden werden empfohlen.
Muñoz-Leyva et al. [14]	2020	„Wesentliche Merkmale von Rebound Pain sind, dass es sich um akute postoperative Schmerzen handelt, welche nach Abklingen der PNB auftreten und klinisch signifikant sind, entweder in Bezug auf die Schmerzintensität, die Auswirkungen auf das psychische Wohlbefinden, die Qualität der Erholung und die Aktivitäten im Alltag."	"Essential characteristics of Rebound-Pain are that it is acute postoperative pain, ensues following resolution of PNB, and is clinically significant, either with regard to the intensity of pain or the impact on psychological well-being, quality of recovery, and activities of daily living."	Review			Rebound-Pain ist ein vorübergehendes Wahrnehmungsphänomen, welches auftritt, wenn sich die sensorische Blockade von RA auflöst und andauernde nozizeptive Reize demaskiert. Glücklicherweise scheint es bei der Mehrheit der Patienten den kumulativen postoperativen Opioidkonsum, die Qualität der Genesung oder die Patientenzufriedenheit nicht signifikant zu beeinflussen und ist nicht mit längerfristigen Folgen wie PPSP verbunden. Rebound-Pain kann daher als Nebenwirkung der RA angesehen werden, die jedoch normalerweise das günstige Nutzen-Risiko-Verhältnis nicht aufhebt. Eine präoperative Schulung ist wesentlich, um angemessene Erwartungen des Patienten zu wecken und ihn über die Bedeutung einer frühzeitigen präventiven Einleitung einer systemischen multimodalen Analgesietherapie aufzuklären. Eine Verlängerung der Wirkungsdauer von PNB mit kontinuierlichen Kathetertechniken oder mit Lokalanästhesie-Zusatzmedikation kann helfen, Rebound-Pain zu lindern, obwohl weitere Forschung erforderlich ist, um dies zu bestätigen.
Barry, Garrett S. et al. [1]	2020	„Ein Übergang von einem gut kontrollierten Schmerz-NRS ≤3, während die Nervenblockade wirkt, zu	"A transition from well-controlled pain NRS ≤3 while the block is working to severe pain NRS ≥7	Retrospektive Kohortenstudie	Patienten mit ambulanten Operationen, die zwischen März 2017 und Februar 2019 eine präoperative PNB erhielten, wurden eingeschlossen.	993 eingeschlossene Patienten	482 (49,6 %) von 972 eingeschlossenen Patienten hatten Rebound-Schmerzen gemäß der Definition. Multivariable Analysen zeigten, dass die Faktoren, die unabhängig voneinander mit Rebound-

		einem starken Schmerz NRS ≥7 innerhalb von 24 Stunden nach Anlage der Nervenblockade.“	within 24 h of block performance.”	(Single-Center)	Rebound-Schmerz wurde definiert als der Übergang von gut kontrolliertem Schmerz (NRS 3), während die Blockade funktioniert, zu starken Schmerzen (NRS 7) innerhalb von 24 h nach der Blockausführung. Patientenbezogene, chirurgische und anästhesiologische Faktoren wurden durch univariate, multivariable und maschinelle Lernmethoden auf Assoziation mit Rebound-Schmerzen analysiert.<br><br>PNB wurde als Lokalanästhetikum definiert, das auf einen Zielnerv aufgebracht wird, unabhängig davon, ob dies hauptsächlich zur Analgesie oder zur chirurgischen Blockade diente (d. h. ob die Blockade mit GA oder Sedierung kombiniert wurde oder nicht).	972 Patienten nach primärer Outcome Analyse	Schmerzen assoziiert waren, jüngeres Alter (OR 0,98; 95 %-KI: 0,97–0,99), weibliches Geschlecht (OR 1,52 [1,15–2,02]), chirurgische Eingriffe waren Knochen (OR 1,82 [1,38–2,40]) und das Fehlen einer perioperativen iv Dexamethason-Gabe (OR 1,78 [1,12–2,83]). Trotz einer hohen Inzidenz von Rebound-Schmerzen gab es hohe Patientenzufriedenheit (83,2 %) und Rückkehr zu täglichen Aktivitäten (96,5 %).<br><br>Rebound-Schmerzen traten bei der Hälfte der Patienten auf und zeigten unabhängige Assoziationen mit Alter, weiblichem Geschlecht, Knochenchirurgie und fehlender intraoperativer Anwendung von i.v. Dexamethason. Bis weitere Forschungsergebnisse verfügbar sind, sollten Kliniker weiterhin präventive Strategien anwenden, insbesondere bei Patienten mit einem höheren Risiko für Rebound-Schmerzen.

<u>Literaturverzeichnis:</u>

1. Barry GS, Bailey JG, Sardinha J et al (2021) Factors associated with rebound pain after peripheral nerve block for ambulatory surgery. Br J Anaesth 126:862–871. https://doi.org/10.1016/j.bja.2020.10.035

2. Dada O, Gonzalez Zacarias A, Ongaigui C et al (2019) Does Rebound Pain after Peripheral Nerve Block for Orthopedic Surgery Impact Postoperative Analgesia and Opioid Consumption? A Narrative Review. Int J Environ Res Public Health 16:3257. https://doi.org/10.3390/ijerph16183257

4. Galos DK, Taormina DP, Crespo A et al (2016) Does Brachial Plexus Blockade Result in Improved Pain Scores After Distal Radius Fracture Fixation? A Randomized Trial. Clin Orthop 474:1247–1254. https://doi.org/10.1007/s11999-016-4735-1

11. Kolarczyk LM, Williams BA (2011) Transient Heat Hyperalgesia During Resolution of Ropivacaine Sciatic Nerve Block in the Rat: Reg Anesth Pain Med 36:220–224.

https://doi.org/10.1097/AAP.0b013e3182176f5a

12. Lavand’homme P (2018) Rebound pain after regional anesthesia in the ambulatory patient. Curr Opin Anaesthesiol 31:679–684. https://doi.org/10.1097/ACO.0000000000000651

14. Muñoz-Leyva F, Cubillos J, Chin KJ (2020) Managing rebound pain after regional anesthesia. Korean J Anesthesiol 73:372–383. https://doi.org/10.4097/kja.20436

15. Nobre LV, Cunha GP, Sousa PCCB de et al (2019) Bloqueio de nervos periféricos e dor rebote: revisão de literatura. Braz J Anesthesiol 69:587–593. https://doi.org/10.1016/j.bjan.2019.05.001

21. Williams B, Bottegal M, Kentor M et al (2007) Rebound Pain Scores as a Function of Femoral Nerve Block Duration After Anterior Cruciate Ligament Reconstruction: Retrospective Analysis of a Prospective, Randomized Clinical Trial. Reg Anesth Pain Med 32:186–192. https://doi.org/10.1016/j.rapm.2006.10.011

22. Williams BA (2012) Forecast for Perineural Analgesia Procedures for Ambulatory Surgery of the Knee, Foot, and Ankle: Applying Patient-centered Paradigm Shifts. Int Anesthesiol Clin 50:126–142. https://doi.org/10.1097/AIA.0b013e31821a00d0
